# A Quality Improvement Initiative of Standard 1 of the British Orthopaedic Association Standards for Trauma (BOAST) for Management of Supracondylar Fractures in Children

**DOI:** 10.7759/cureus.96635

**Published:** 2025-11-11

**Authors:** Muhammad Rizwan Umer, Shahmeen Rasul, Mohamed K Abouelsadat, Shenouda Shehata Abdelmesih, Taher Mohammed, Ahmed S Ibrahim, Mohammed Elfatih Elbadri, Abdelrahman Sahnon Abaker Sahnon, Mariam Melkumian, Lubna Rehman

**Affiliations:** 1 Trauma and Orthopaedics, Royal Sussex County Hospital, Brighton, GBR; 2 Trauma and Orthopaedics, Royal Free Hospital, London, GBR; 3 Vascular Surgery, Royal Free Hospital, London, GBR; 4 Orthopaedics and Traumatology, Khoula Hospital, Muscat, OMN; 5 Medical Education, StepExcel Boards Academy, Skokie, USA; 6 Orthopaedics and Traumatology, Nile University, Khartoum, SDN; 7 Orthopaedics, Khorfakkan Hospital, Sharjah, ARE; 8 General Practice, Medical Council of Ireland, Dublin, IRL; 9 General Surgery, Royal College of Surgeons of Edinburgh, Edinburgh, GBR; 10 Research, StepExcel Boards Academy, Skokie, USA; 11 General Medicine, Dow University of Health Sciences, Civil Hospital Karachi, Karachi, PAK

**Keywords:** audit, boast guidelines, neurovascular assessment, orthopaedics, pediatric trauma, supracondylar fracture

## Abstract

Background: Supracondylar humeral fractures are the most common elbow injuries in children and carry a high risk of neurovascular compromise. The British Orthopaedic Association Standards for Trauma (BOAST) recommend thorough documentation of vascular and nerve assessment in every case.

Objective: To evaluate compliance with BOAST Standard 1 for neurovascular documentation in paediatric supracondylar fractures at a large NHS hospital trust.

Methods: A retrospective cohort audit of 44 patients between January and May 2022 was performed. Neurovascular documentation, imaging practices, and adherence to BOAST criteria were assessed from e-Trauma and PACS (Picture Archiving and Communication System) records. Statistical analysis was carried out using IBM SPSS Statistics for Windows, version 24 (IBM Corp., Armonk, NY, USA).

Results: Forty-four paediatric patients with supracondylar fractures were included (mean age 7.4 years, 61% male). Extension-type fractures accounted for 93%, with Gartland Types I, II, and III representing 30% (n=13), 43% (n=19), and 27% (n=12), respectively. Only 10% (n=4) of patients had detailed nerve function documented, while 40 (90%) were recorded as “neurovascular intact.” Radial artery status was missing in 25 (57%) cases, and capillary refill time was not recorded in 14 (32%). Post-cast X-rays were obtained in 29 (66%) patients, but 12 (28%) were discharged in a cast without imaging. Overall, compliance with BOAST Standard 1 was poor.

Conclusion: Compliance with BOAST Standard 1 was poor, especially in the documentation of nerve and vascular status. This represents a significant patient safety concern. Educational interventions and structured proformas are required to improve adherence and should be followed by re-audit.

## Introduction

Supracondylar humeral fractures represent the most frequent type of elbow fracture in children, accounting for up to 60% of elbow injuries in the paediatric population [1]. These injuries typically occur following a fall onto an outstretched hand and are most often extension-type fractures. The anatomical proximity of the brachial artery and major peripheral nerves makes these fractures clinically significant because of the high risk of neurovascular injury [2]. The Gartland classification system is widely used to grade supracondylar fractures based on displacement and stability [3]. Type I injuries are nondisplaced, Type II are displaced with an intact posterior cortex, and Type III are completely displaced. Accurate classification guides management, from conservative immobilisation to urgent operative fixation. However, regardless of fracture type, the cornerstone of management is a meticulous neurovascular examination at presentation.

The British Orthopaedic Association Standards for Trauma (BOAST) provide clear guidelines to standardise care. Standard 1 of the BOAST guidelines mandates that every child with a supracondylar fracture should have documented assessment of radial pulse, capillary refill time, and individual motor/sensory testing of the radial, median (anterior interosseous nerve, AIN), and ulnar nerves [4]. Adherence to these recommendations ensures early detection of vascular compromise or nerve palsy, both of which may have long-term consequences if missed. Despite well-established guidelines, adherence in clinical practice remains inconsistent. Previous audits have highlighted gaps in documentation and variability in imaging practices, which may compromise patient safety [5,6]. Addressing these gaps is essential to improve patient safety, standardise care, and reduce medico-legal risk. This audit, therefore, directly evaluates compliance with BOAST Standard 1 to guide targeted quality-improvement interventions. Therefore, this study was designed to assess compliance with BOAST Standard 1 within a large NHS Trust, identifying deficiencies and informing strategies for quality improvement.

Aim of the study

The primary aim of this audit was to evaluate compliance with BOAST Standard 1 for neurovascular documentation in children with supracondylar humeral fractures. Secondary objectives included assessing imaging practices, identifying gaps in documentation, and informing institutional strategies to enhance patient safety.

## Materials and methods

Study design and setting

A retrospective cohort audit was conducted at London North West University Healthcare NHS Trust. All paediatric patients (≤16 years) presenting with supracondylar fractures between January 1, 2022, and May 31, 2022, were included.

Case sampling and eligibility criteria

All paediatric cases of supracondylar humeral fractures were identified from the hospital’s e-Trauma database using the keyword “supracondylar.” Patients were eligible for inclusion if they were aged 16 years or younger, had radiographically confirmed supracondylar fractures, and presented between January 1 and May 31, 2022. Cases were excluded if the fracture was open or pathological, if clinical documentation was incomplete, or if the patient had been transferred from another hospital. All cases meeting these criteria during the study period were included, representing consecutive admissions obtained through convenience sampling.

Data collection

Data for this audit were obtained from the hospital’s e-Trauma database by using a structured checklist designed according to BOAST Standard 1 parameters, which records all trauma presentations. For each identified case of supracondylar fracture, patient notes were reviewed to determine whether the neurovascular assessment was documented according to BOAST Standard 1. Specifically, this included the presence or absence of radial pulse, capillary refill time (CRT), and the function of the radial, median (anterior interosseous nerve, AIN), and ulnar nerves, which were evaluated through a standard neurovascular examination. The radial pulse was palpated at the wrist to assess its presence and quality, while CRT was measured at the fingertip or nail bed by applying pressure and observing the time for colour to return, with less than two seconds considered normal. Radial nerve function was assessed by asking the child to extend the wrist and fingers, whereas AIN function was tested by the ability to form an “OK sign” through flexion of the thumb interphalangeal and index distal interphalangeal joints. Ulnar nerve function was examined by finger abduction, adduction, or crossing of the index and middle fingers [7]. Radiographs stored in the hospital’s Picture Archiving and Communication System (PACS) were examined to classify the fractures using the Gartland system and to assess whether post-cast images were obtained. The Gartland classification system is commonly used to describe supracondylar fractures of the humerus in children based on radiographic appearance and fracture displacement. Type I fractures are non-displaced, where the fracture line is visible on radiographs but there is no displacement, and the anterior humeral line still intersects the capitellum. In Type II fractures, there is partial displacement of the fracture with an intact posterior cortex. Radiographically, the anterior humeral line is shifted anterior to the capitellum due to posterior angulation of the distal fragment. Type III fractures are characterised by complete displacement with no cortical contact; the distal fragment is completely separated and typically moves posteriorly. Type IV fractures indicate complete disruption of the periosteum and are unstable in both flexion and extension views, suggesting multidirectional instability. This classification helps guide treatment decisions and predict potential complications [8]. Cases discharged without post-cast imaging were flagged for further analysis. Demographic variables such as age, sex, and mechanism of injury were also collected to provide descriptive context for the cohort.

Ethical considerations

The Hospital NHS Trust Clinical Audit Department reviewed the study and concluded that it does not qualify as human research, as it was conducted solely for the purpose of service evaluation and quality improvement. No patient identifiers were collected.

BOAST guideline criteria

The BOAST guidelines outline clear standards for the management of displaced supracondylar fractures in children [9]. Management of supracondylar fractures in children involves a structured approach to ensure safety and optimal outcomes. On admission and prior to surgery, a thorough limb assessment must be performed, documenting the radial pulse, capillary refill, and the function of the radial, median, and ulnar nerves. Surgery should ideally be performed on the same day as the injury, though nighttime procedures are only necessary if there are urgent indications. Urgent surgery is required in cases where there is no palpable pulse, signs of poor perfusion, an open fracture, or threatened skin integrity. Typically, vascular status improves after fracture reduction, and exploration of the artery is not necessary if the hand remains well perfused, even in the absence of a detectable pulse. For fixation, at least two K-wires crossing the cortex should be used. Crossed wires offer better stability, while lateral divergent wires reduce the risk of ulnar nerve injury. If a medial wire is placed, protective measures for the ulnar nerve must be employed and properly documented. A wire size of 2 mm is preferred, and intraoperative stability and alignment must be assessed and recorded. In cases of persistent ischemia after reduction, the brachial artery should be explored by a surgeon skilled in vascular repair. Postoperatively, neurovascular monitoring must continue until vascular compromise or compartment syndrome is definitively ruled out. Any suspicion of nerve injury should prompt consultant review. Signs of deteriorating perfusion may indicate compartment syndrome and require immediate reassessment and intervention. The operating surgeon is responsible for specifying whether postoperative X-rays are needed and when the K-wires should be removed. While routine long-term follow-up is not typically necessary, any clear indications for further review must be documented. Together, these parameters form the benchmark for appropriate neurovascular assessment in this clinical setting.

Data analysis

Data were analysed using IBM SPSS Statistics for Windows, Version 24.0 (IBM Corp., Armonk, NY, USA) [10]. Categorical variables were summarised as frequencies and percentages; continuous variables as means ± standard deviation. The Chi-square test was used to compare categorical documentation rates, as all variables were nominal. Fisher’s exact test was applied when expected cell counts were <5. Effect sizes (Cramer’s V) and 95% confidence intervals (CIs) were calculated to quantify the strength of associations. A two-tailed p-value <0.05 was considered statistically significant.

## Results

Demographics

**Figure 1:**
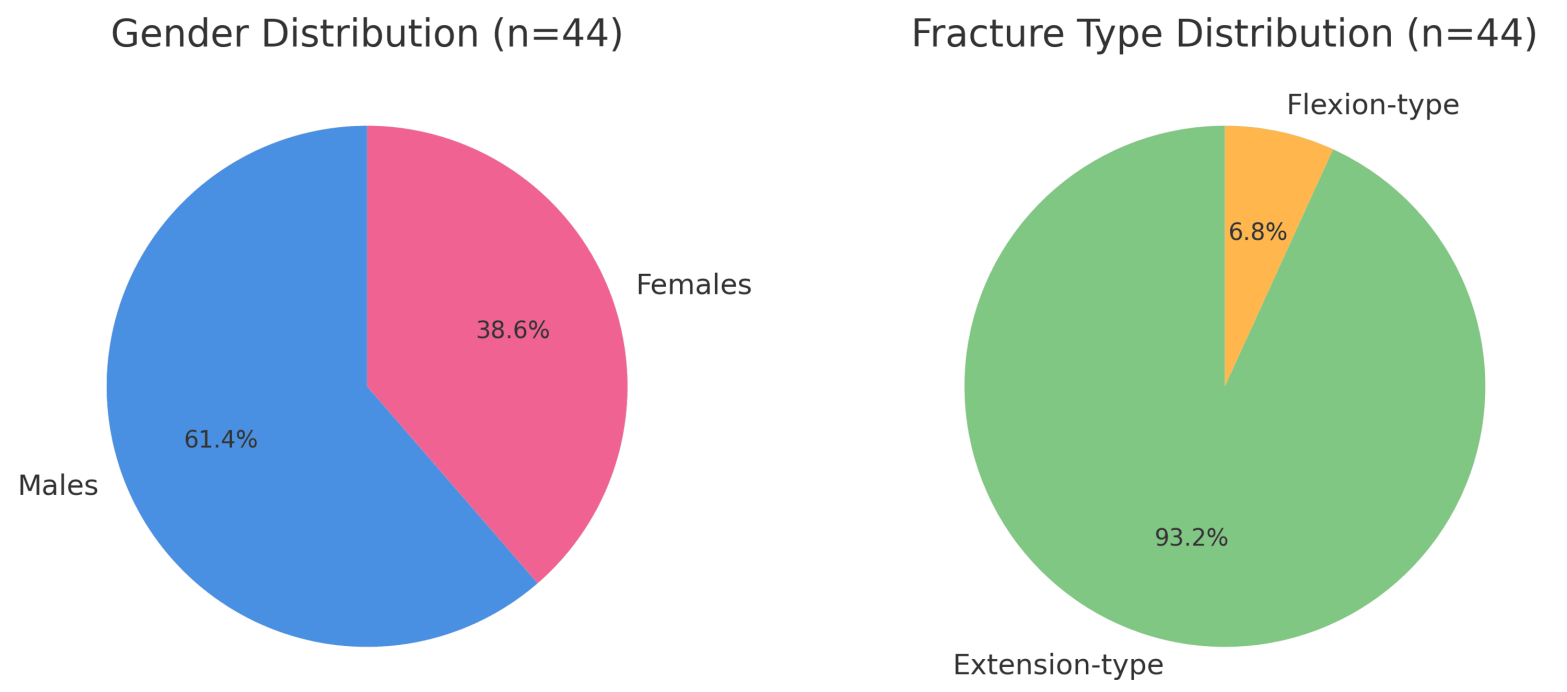
Demographic and fracture type distribution of paediatric supracondylar humerus fractures

A total of 44 paediatric patients with supracondylar humeral fractures were analysed. The mean age at presentation was 7.4 years (SD ±2.1; range 3-12 years). Males accounted for 27 (61.4%) cases and females for 17 (38.6%), giving a male-to-female ratio of 1.6:1. The predominance of extension-type injuries was striking, with 41 (93.2%) patients sustaining extension-type fractures compared to only 3 (6.8%) with flexion-type injuries, as per Figure 1.

Fracture classification

**Figure 2:**
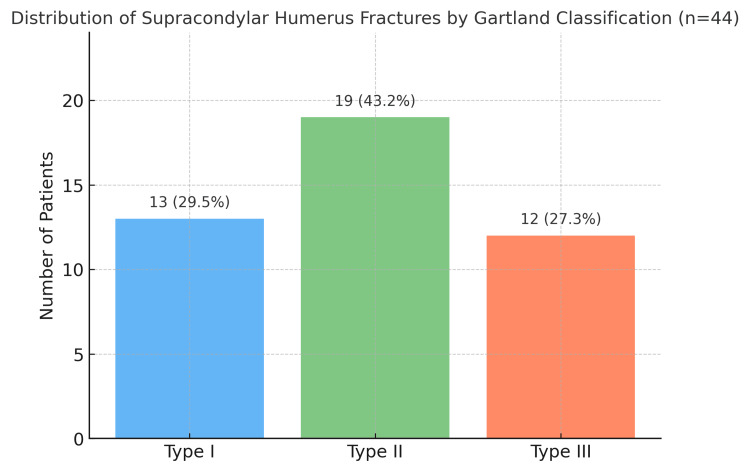
Distribution of supracondylar humerus fractures by Gartland classification

Based on the Gartland classification, Type II fractures were the most frequent, observed in 19 (43.2%) cases, followed by Type I in 13 (29.5%) and Type III in 12 (27.3%). This distribution reflects the common clinical pattern where incomplete displacement (Type II) predominates, though a significant proportion still presented with fully displaced (Type III) injuries requiring more intensive management, as shown in Figure 2.

Neurovascular documentation

Compliance with BOAST Standard 1 for neurovascular documentation was generally suboptimal. Detailed assessment of the radial, median (AIN), and ulnar nerves was recorded in only 4 (9.1%) cases, whereas 40 (90.9%) were broadly documented as “neurovascularly intact.” This difference was statistically significant (χ² = 28.9, p < 0.001), with a large effect size (Cramer’s V = 0.81), indicating substantial under-documentation. Radial artery status was omitted in 25 (56.8%) patients, reflecting substandard practice; however, this difference did not reach statistical significance (χ² = 0.36, p = 0.55, Cramer’s V = 0.09, small effect). Capillary refill time (CRT) was documented in 30 (68.2%) cases and omitted in 14 (31.8%), a significant finding (χ² = 5.82, p = 0.02, Cramer’s V = 0.36, medium effect), although CRT was not consistently cross-referenced with radial pulse, limiting its interpretive value. Post-cast X-rays were performed in 29 (66%) cases and omitted in 15 (34%), a statistically significant difference (χ² = 4.45, p = 0.03, Cramer’s V = 0.32, medium effect). Overall, chi-square analysis and effect size evaluation confirmed significant under-documentation across most neurovascular and imaging parameters, highlighting that comprehensive assessment was incomplete in the majority of patients. A structured summary of neurovascular and imaging compliance, including effect sizes, is presented in Table 1.

**Table 1:** Compliance with BOAST standard 1 for supracondylar fractures in children CRT: capillary refill time; AIN: anterior interosseous nerve (branch of the median nerve); BOAST: British Orthopaedic Association Standards for Trauma Post-cast X-ray: Radiograph performed after application of the cast to confirm alignment Documented (%): Percentage of cases where the parameter was recorded in notes Not documented (%): Percentage of cases where the parameter was not recorded in notes Data analysed using the Chi-square (χ²) test for categorical variables; a p-value <0.05 was considered statistically significant; * indicates statistical significance

Parameter	Documented n (%)	Not documented n (%)	χ² Value	p-value	Cramer’s V	Effect size interpretation
Radial pulse	19 (43%)	25 (57%)	0.36	0.55	0.09	Small
Capillary refill time (CRT)	30 (68%)	14 (32%)	5.82	0.02*	0.36	Medium
Radial nerve	4 (10%)	40 (90%)	28.9	<0.001*	0.81	Large
Median (AIN) nerve	4 (10%)	40 (90%)	28.9	<0.001*	0.81	Large
Ulnar nerve	4 (10%)	40 (90%)	28.9	<0.001*	0.81	Large
Post-cast X-ray performed	29 (66%)	15 (34%)	4.45	0.03*	0.32	Medium

Imaging practices

Post-cast radiographs, intended to confirm maintenance of alignment and reduction adequacy, were performed in 29 (65.9%) patients, while 15 (34.1%) did not undergo post-cast imaging. The difference between documented and non-documented imaging was statistically significant (χ² = 4.45, p = 0.03). Among the non-imaged group, 3 (6.8%) patients were managed with collar-and-cuff immobilisation or taken directly for operative fixation, and 12 (27.3%) were discharged with a cast without radiological confirmation of reduction. This represents a significant deviation from recommended practice and raises concerns about the possibility of undetected loss of reduction, especially in those discharged without imaging.

Compliance summary

Overall adherence to BOAST Standard 1 was suboptimal. Detailed documentation of individual nerve function was rarely performed, vascular status was inconsistently assessed, and post-cast imaging was not universally obtained. These gaps highlight a significant shortfall in meeting national standards of care.

## Discussion

This audit demonstrated significant deficiencies in compliance with BOAST Standard 1, particularly in the documentation of individual nerve function and radial pulse assessment. Detailed neurovascular assessments were rarely recorded, and capillary refill time was inconsistently cross-checked with radial pulse. Post-cast imaging was performed in most cases, but was not universally documented. These findings highlight a critical area for quality improvement within paediatric trauma care and underscore the need for standardised documentation practices. Although these injuries are common, detailed documentation of individual nerve function was recorded in only 4 (9.1%) cases, while the remaining 40 (90.9%) relied on the nonspecific phrase “Neurovascularly intact.” Such generalised recording falls short of national standards, which require individual assessment of the radial, median (including the anterior interosseous branch), and ulnar nerves, all of which are at risk in this fracture pattern [11]. The medico-legal importance of documentation cannot be overstated, as the principle “if it is not documented, it did not happen” remains firmly upheld in litigation [12]. Vascular documentation was similarly incomplete. Radial artery status was omitted in more than half of patients (56.8%), despite the well-recognised consequences of missed vascular compromise, such as Volkmann’s ischemic contracture, which carries lifelong morbidity [13].

Although the incidence of vascular injury is relatively low (5-10%), omission of arterial documentation represents a patient safety concern. Capillary refill time (CRT) was more consistently recorded (68.2%) but remained absent in nearly one-third of cases. This pattern mirrors the findings of Argyriou et al., who reported similarly incomplete vascular assessments in paediatric trauma, emphasising the ongoing gap between best practice standards and real-world documentation [14]. Comparison with other centres demonstrates that structured tools improve compliance. Studies by Faraz et al. and Cao et al. found that introducing standardised proformas or electronic checklists significantly increased neurovascular documentation quality and improved patient outcomes [15,16]. In contrast, the present audit shows that in the absence of such structured systems, documentation rates remain unacceptably low. Post-cast radiographic evaluation revealed a further gap in practice. Although 29 (65.9%) patients underwent confirmatory imaging, 12 (27.3%) were discharged in a cast without post-reduction radiographs. This deviates from accepted standards, especially for Gartland Type II and III fractures, where the risk of malalignment or loss of reduction is high. Our findings align with Cheng et al. and Zusman et al., who both advocate routine post-cast imaging to detect early displacement and reduce the risk of revision surgery [17,18]. Compared to those studies, our compliance rate appears lower, raising concerns particularly in teaching hospital settings where reductions are frequently performed by junior clinicians. In such contexts, objective radiographic confirmation is crucial to safeguard care quality.

Based on these findings, several interventions are recommended. First, the introduction of a structured neurovascular documentation proforma integrated into the electronic record could standardise assessment and reduce omissions. Second, targeted educational sessions for emergency and orthopaedic junior staff may improve awareness and competence in neurovascular documentation. Finally, implementation of a regular re-audit cycle every six months would allow monitoring of sustained improvement and identification of persistent gaps in practice. Common barriers to full adherence may include time constraints in busy emergency settings, variability in staff experience and confidence, and the absence of standardised templates within the electronic documentation system. Addressing these systemic factors is essential to achieving consistent compliance with BOAST guidelines and improving patient safety.

This audit was limited by its retrospective design, relying on existing documentation rather than observed clinical practice, introducing potential documentation bias. The relatively small sample size (44 patients) and single-centre scope may also limit the generalizability of the findings. Additionally, convenience sampling of consecutive admissions may introduce selection bias. Nevertheless, this study establishes a clear baseline for future quality-improvement initiatives within paediatric trauma care. A re-audit following the implementation of an electronic checklist is planned to complete the audit cycle and evaluate improvement in BOAST compliance rates. This will provide objective data on the effectiveness of the proposed interventions and inform ongoing efforts to enhance patient safety and clinical documentation standards.

## Conclusions

This audit revealed that compliance with BOAST Standard 1 in the management of paediatric supracondylar fractures was substantially below recommended levels, particularly regarding documentation of individual nerve function and radial pulse assessment. Omissions of these critical elements pose direct risks to patient safety and reduce the reliability of clinical records. Inconsistent acquisition of post-cast radiographs further underscores gaps in adherence to best practice, potentially compromising the detection of malalignment and optimal outcomes. Addressing these deficiencies will require structured educational programs, standardised documentation templates, and reinforcement of BOAST guidelines in routine clinical practice. Subsequent re-audit will be essential to ensure sustained improvement and safe, guideline-compliant care for children with supracondylar fractures.
